# INDIVIDUAL AND RELATION-INFERRED SELF-EFFICACY FOR PHYSICAL ACTIVITY INCREASE IN OLDER ADULT COUPLES

**DOI:** 10.1093/geroni/igae098.4222

**Published:** 2024-12-31

**Authors:** Elizabeth Zambrano Garza, Tiana Broen, Maureen Ashe, Kenneth Madden, Denis Gerstorf, Christiane Hoppmann

**Affiliations:** University of British Columbia, Vancouver, British Columbia, Canada; University of Colorado Colorado Springs, Colorado Springs, Colorado, United States; University of British Columbia, Vancouver, British Columbia, Canada; University of British Columbia, Vancouver, British Columbia, Canada; Humboldt University Berlin, Berlin, Berlin, Germany; University of British Columbia, Vancouver, British Columbia, Canada

## Abstract

Older adults encounter daily problems which may hinder them to engage in physical activity. Individual self-efficacy has been positively associated with physical activity, but it does not consider the social world we live in; relation-inferred self-efficacy does, as it captures one’s perception about whether a close other believes in their abilities to engage in these behaviors. With a sample of 110 community-dwelling older adult couples who wore accelerometers and reported daily ratings of problems for up to 7 days, this project analyzes associations of both types of self-efficacy and daily problems with physical activity (MVPA and steps). Assuming that both modalities of self-efficacy may be particularly useful to motivate individuals, interactions of self-efficacy and daily problems were examined. We controlled for well-established associations with physical activity (age, gender, and physical limitations). Daily problems were not associated with physical activity. When modelled separately, both types of self-efficacy were associated with more physical activity; when modelled together, only relation-inferred self-efficacy was significant. Unfortunately, there were no significant interaction effects. Findings replicate previous evidence and shed light on the importance of social context for physical activity engagement in older adulthood.

